# Brd4 and HEXIM1: Multiple Roles in P-TEFb Regulation and Cancer

**DOI:** 10.1155/2014/232870

**Published:** 2014-01-29

**Authors:** Ruichuan Chen, Jasper H. N. Yik, Qiao Jing Lew, Sheng-Hao Chao

**Affiliations:** ^1^State Key Laboratory of Cellular Stress Biology, School of Life Sciences, Xiamen University, Xiamen, Fujian 361005, China; ^2^Department of Orthopaedic Surgery, Lawrence J. Ellison Musculoskeletal Research Center, University of California at Davis Medical Center, Sacramento, CA 95817, USA; ^3^Expression Engineering Group, Bioprocessing Technology Institute, A∗STAR (Agency for Science, Technology and Research), 20 Biopolis Way, No. 06-01, Singapore 138668; ^4^Department of Microbiology, National University of Singapore, Block MD4, 5 Science Drive 2, Singapore 117597

## Abstract

Bromodomain-containing protein 4 (Brd4) and hexamethylene bisacetamide (HMBA) inducible protein 1 (HEXIM1) are two opposing regulators of the positive transcription elongation factor b (P-TEFb), which is the master modulator of RNA polymerase II during transcriptional elongation. While Brd4 recruits P-TEFb to promoter-proximal chromatins to activate transcription, HEXIM1 sequesters P-TEFb into an inactive complex containing the 7SK small nuclear RNA. Besides regulating P-TEFb's transcriptional activity, recent evidence demonstrates that both Brd4 and HEXIM1 also play novel roles in cell cycle progression and tumorigenesis. Here we will discuss the current knowledge on Brd4 and HEXIM1 and their implication as novel therapeutic options against cancer.

## 1. Introduction

Transcriptional regulation is a fundamental process for converting the genetic codes into RNA synthesis for proper cellular functions in an organism. The main cellular machinery for transcribing all protein-coding genes is the RNA polymerase II (RNAPII). Due to the enormous numbers of different genes to be coordinately expressed at any given time, the transcriptional activity of RNAPII is subjected to meticulous modulation by a myriad of regulators. One of the most significant RNAPII regulators identified to date is the positive transcriptional elongation factor b (P-TEFb). P-TEFb controls the transition of RNAPII from promoter proximal pausing to productive elongation for efficient full-length mRNA synthesis [1, 2]. At the early phase of mRNA synthesis shortly after the initiation of transcription, the progression of RNAPII is paused near the transcriptional start site by the concerted actions of the negative elongation factor (NELF) [3], DRB (5,6-dichloro-1-b-D-ribofuranosyl-benzimidazole) sensitivity-inducing factor (DSIF) [4], and Gdown1 [5]. In order to release the paused RNAPII, P-TEFb is recruited to the promoter, where its kinase activity phosphorylates the C-terminal domain (CTD) of RNAPII and the negative elongation factors NELF and DSIF [6, 7]. These phosphorylation events are believed to induce conformational changes that allow RNAPII to escape promoter proximal pausing and enter the productive phase of transcriptional elongation. More importantly, the pausing of RNAPII serves as a rate-limiting step for transcriptional regulation, especially for inducible genes, such as those induced by stress and inflammatory responses [8, 9]. Cells treated with flavopiridol, the most potent and selective P-TEFb inhibiting compound, result in inhibition of 60–70% of RNAPII transcription [10, 11]. Furthermore, genome-wide studies find that RNAPII occupies the promoters of most protein-coding genes in *Drosophila* and human embryonic stem cells without entering into productive elongation [12–16]. Therefore, P-TEFb plays a critical role in RNAPII regulation and has a broad impact on global gene expression patterns that affect almost every aspect of cellular functions.

Besides controlling the expression of cellular genes, P-TEFb is also required for transcriptional regulation of a number of viruses. The best-known example is the human immunodeficiency virus (HIV). Mathews and coworkers detected two classes of HIVpromoted cytoplasmic RNAs, a fulllength transcript, and a short transcript ending 55–59 nucleotides from the start site of transcription [17]. This observation clearly implies that transcription of HIV may also be regulated at the elongation stage. Later studies demonstrate that the HIV Tat protein recruits P-TEFb to the viral promoter and triggers the transition of RNAPII into productive elongation, resulting in the generation of full-length viral transcripts [18–20].

**Figure 1:**
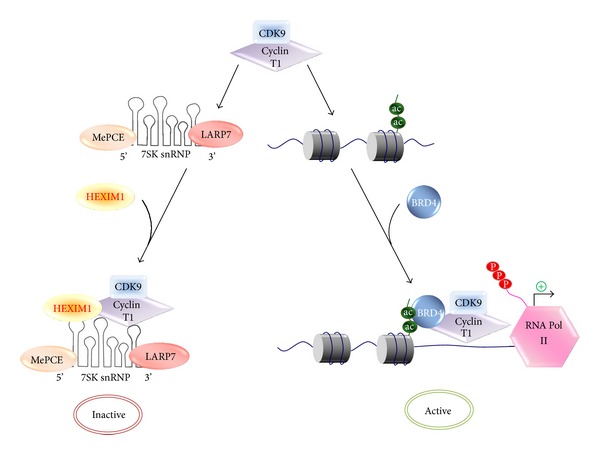
Regulation of P-TEFb activity by its positive regulator HEXIM1 and negative regulator Brd4.

P-TEFb is composed of cyclin-dependent kinase 9 (CDK9) and its regulatory cyclin partner cyclin T1 [21, 22]. The Ser2 of the RNA Polymerase II CTD repeat (i.e., YSPTSPS) has been identified as the P-TEFb phosphorylation site during elongation [23, 24]. CDK12 is also a CTD Ser2 kinase; however, it is not within the scope of this review [25]. CDK9 exists as two isoforms, a major 42 kDa form and a minor 55 kDa form [26]. Besides cyclin T1, minor CDK9-associated cyclins, such as T2a, T2b, and K, are also present but at much lower levels in many cell types [27, 28]. Given the important roles of P-TEFb in RNAPII-dependent transcription, P-TEFb's activity is constantly being modulated through dynamic association with positive and negative regulators. Among these factors, hexamethylene bisacetamide (HMBA) inducible protein 1 (HEXIM1) and bromodomain-containing protein 4 (Brd4) are the two major regulators of P-TEFb. In log-phase HeLa cells, roughly half of the P-TEFb is sequestered into an inactive complex containing the kinase inhibitor HEXIM1 [29, 30] and other auxiliary proteins, MePCE [31], and LARP7 [32] held together by the non-coding 7SK small nuclear RNA (snRNA) (Figure 1). HEXIM1 exerts its inhibitory function on P-TEFb only when associated with the 7SK snRNA, while neither 7SK nor HEXIM1 alone instigates any effects [29, 33]. It has been proposed that association of the 7SK snRNA with HEXIM1 leads to a conformational change that renders the cyclin T1-binding domain of HEXIM1 accessible for P-TEFb binding [34]. Besides the inactive pool of P-TEFb, the remaining half of P-TEFb is transcriptionally active and bound to Brd4. Brd4 functions to recruit P-TEFb to active promoter through its affinity to acetylated histones [35, 36] (Figure 1). The distribution of P-TEFb between the active and inactive complexes is a dynamic process that can be rapidly changed in response to cellular stress and transcriptional demand. The regulation of P-TEFb by Brd4 and HEXIM1 has been extensively reviewed elsewhere [37, 38]. In this review, we will focus on the recent findings on Brd4/HEXIM1 with respect to their newly discovered roles in cell cycle progression and cancer.

## 2. Brd4

During eukaryotic transcription, the epigenetic information packaged in chromatin is interpreted by “reader” proteins. These proteins contain specialized domains for recognizing the vocabularies of histone codes that are composed of combinatorial histone modifications [39]. Histone acetylation, for instance, is specifically recognized by protein module termed bromodomain [40]. In human, there are a total of 61 different bromodomains. These bromodomains are evolutionarily conserved and present in 46 distinct proteins, which belong to eight families: histone acetyltransferases, ATP-dependent chromatin-remodeling complexes, helicases, methyltransferases, transcriptional coactivators, transcriptional mediators, nuclear-scaffolding proteins, and the BET family proteins [41, 42].

Brd4 is a ubiquitously expressed nuclear protein that belongs to the BET protein family. Brd4 contains two N-terminal tandem bromodomains (BDI and BDII) and an extraterminal domain (ET) [43]. The ET domain functions as a motif that interacts with WHSC1L1 (Wolf-Hirschhorn syndrome candidate 1-like 1 or NSD3) for the regulation of H3K36 methylation [44]. Early reports indicated that the bromodomains of Brd4 are essential for its binding to chromatin via acetylated histones [45, 46]. A recent study with systematic peptide array screening combined with crystallography further revealed that the first bromodomain BD1 of Brd4 contributes mostly for its association with acetylated chromatin by simultaneous binding and recognition of diverse diacetyl-containing histones [41].

### 2.1. The Dual Faces of Brd4: Cell Cycle Control and Transcriptional Regulation

Consistent with its chromatin targeting nature, Brd4 was originally identified as a mitotic chromosome associated protein (MCAP) as it was found persistently associated with acetylated chromosomes during mitosis in a number of cell lines [45, 46]. Later studies demonstrated that this association is critical for the rapid expression of early G1 genes upon exiting mitosis [47–50]. Although the mechanism is still unclear, Brd4 has been proposed to play an important role in transmitting the epigenetic memory across cell division by bookmarking the transcriptionally active genes [49, 51, 52]. Besides the bookmarking function, the association of Brd4 with mitotic chromosome is also important for Brd4's function in maintaining chromatin compaction [50, 53] and in facilitating the even segregation of viral DNAs into daughter cells during cell division [54].

Distinct from the other BET proteins, Brd4 contains a unique PID domain at its extreme C-terminus for interacting with P-TEFb [55]. Increasing evidence indicates that for the expression of many signal-inducible genes, the rate-limiting step is not transcription initiation but elongation [56]. After initiation is accomplished, RNAPII is poised at the promoter-proximal regions, and signal-induced activation and recruitment of P-TEFb to these regions convert the stalled RNAPII into productive elongation mode [57, 58].

In cell, P-TEFb is tightly regulated by being sequestrated in the nucleoplasma as an inactive ribonucleoprotein complex that contains the 7SK snRNA, HEXIM1, LARP7, and MePCE [59, 60] (Figure 1). Upon stimulation, P-TEFb is liberated from the inactive 7SK complex by the dephosphorylation of CDK9 T-loop through the concerted actions of PP2B and PP1*α* (protein phosphatase 2B and 1*α*) pathways or by the phosphorylation of HEXIM1 through PI3D/Akt or PKC kinase signaling pathways [61–63]. Subsequently, the liberated P-TEFb is recruited by Brd4 onto promoter-proximal region to modulate the processivity of RNAPII [29, 35, 36, 61, 64]. In line with its role in recruiting P-TEFb for stimulation of transcriptional elongation, Brd4 has been shown to be indispensable for cell proliferation [47–49], as well as the integration and transcription of HIV-1 [65–70], inflammatory response [8, 71, 72], cardiac hypertrophy [73, 74], and DNA damage repair [75, 76].

### 2.2. Brd4, a Novel Drug Target for Cancer Therapy

Accumulating studies have revealed the critical roles of Brd4 in cancer development [77, 78]. The first clue linking Brd4 with cancer was the finding of Brd4-*NUT* fusion oncogene which was recognized as an important mechanism in *NUT* midline carcinoma (NMC), an aggressive form of squamous carcinoma [79, 80]. NMC is genetically defined by the chromosomal rearrangements of the gene *NUT* (*C15* or *f55*). In about 75% of NMCs, most of the *NUT* gene's coding region on chromosome 15q14 is fused with Brd4 [81–83]. Besides the chromosomal rearrangement-induced *NUT* midline carcinomas, other studies have also indicated that Brd4 may contribute to cancer development through different mechanisms. For example, deleting the proline-rich region of Brd4 could promote epithelial-to-mesenchymal transition and stem cell-like conversion [84, 85]. The interaction of the first bromodomain (BD1) of Brd4 with acetylated NF-*κ*B/RelA leads to constitutively active NF-*κ*B that enhances cancer cell proliferation [86]. Moreover, a study using shRNA library targeting 243 known chromatin regulators identified Brd4 as a required factor for the maintenance of acute myeloid leukemia (AML). Knockdown of Brd4 exhibited a robust antileukemic activity against AML *in vitro* and *in vivo* [87, 88]. Other recent studies with small molecule inhibitors of the BET proteins, such as JQ1 and I-BET 151, revealed the critical role of Brd4 in the development of several hematopoietic and somatic cancers, such as Burkitt's lymphoma, multiple myeloma [88–91], melanoma [92], colon [93], and breast cancer [84]. The mode of action of the BET inhibitors may be, at least in part, due to the inhibition of transcription of the oncogene, C-MYC [89–91]. A recent study shows that treatment with JQ1 results in preferential loss of Brd4 at super-enhancers and consequent transcription elongation defects that preferentially impacted genes with super-enhancers, including MYC [94]. Accordingly, small molecule inhibitors targeting Brd4 have been proven to be a promising drug for cancer therapy [77, 95].

### 2.3. The Role Switching of Brd4: From Chromatin Targeting to Transcriptional Regulation

The diverse biological roles of Brd4 have been proposed to rely on its functional transition between chromatin targeting and transcriptional regulation [43, 51]. Consistent with this notion, our recent study showed that, besides P-TEFb, the availability of Brd4 is also highly regulated [64]. Surveys of a variety of cell lines show that almost all Brd4 is bound to chromatin in interphase and there is limited chromatin-free Brd4 available in the unstimulated state. Upon stress induction (such as UV or HMBA treatment), Brd4 is released from chromatin and subsequently interacts and recruits signal-activated P-TEFb to enhance transcriptional elongation of inducible genes [64]. Intriguingly, stress treatment also triggers global deacetylation of acetylated-lysine 5 and 8 of nucleosomal histone H4 (H4K5ac/K8ac) [64]. In our recent study, these two acetylated sites are identified as the Brd4 binding sites (unpublished data), suggesting that the signal-controlled dynamic modification of nucleosomal histone is important for Brd4's role transition from chromatin targeting to transcriptional regulation.

**Figure 2:**
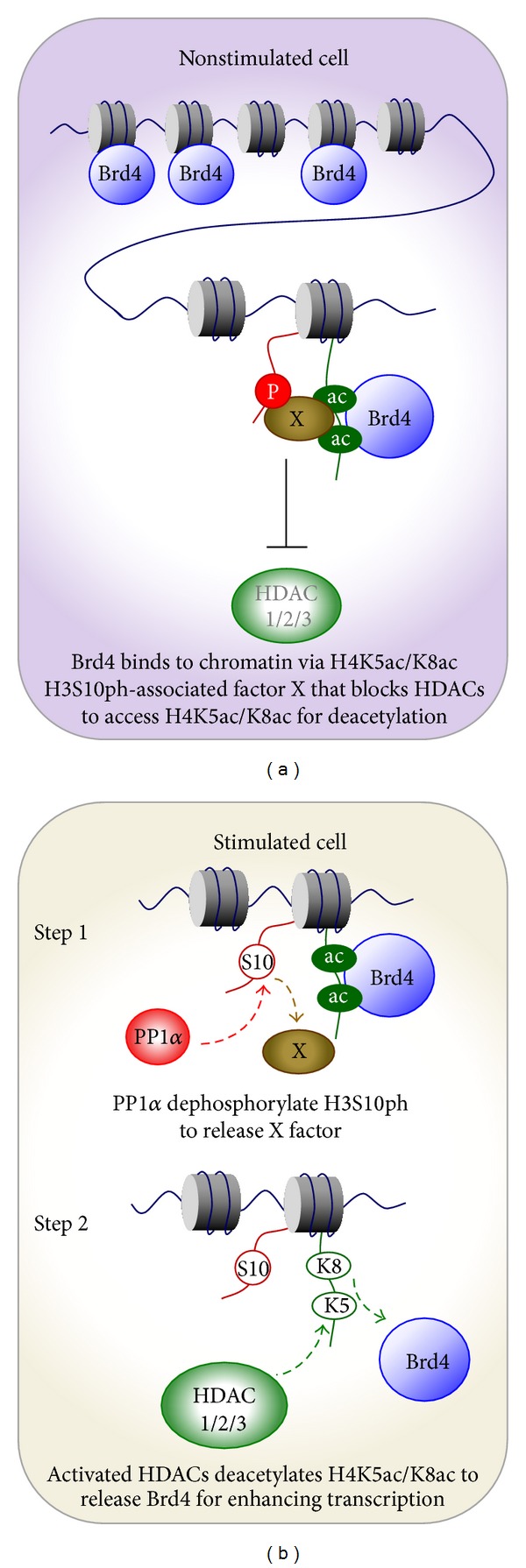
Regulation of the association between Brd4 and chromatins in the nonstimulated (a) and stimulated (b) cells.

Since the release of chromatin-bound Brd4 correlates with the signal-induced deacetylation of Brd4 binding sites H4k5ac/K8ac, the epigenetic regulation of Brd4's role switching apparently could simply be the deacetylation of Brd4's binding sites by histone deacetylases (HDACs). Through *in vivo* experiments and *in vitro* biochemical assays, we unexpectedly find that both PP1*α* and HDACs (HDAC1, 2, and 3 of class I HDAC) signaling pathways are essential for releasing chromatin-bound Brd4. Moreover, this releasing process relies on *trans-*histone crosstalk between H3S10ph (phosphorylated-serine 10 of histone H3) and H4K5ac/K8ac, which connects PP1*α* and HDACs to control the functional transition of Brd4 (Figure 2). In the nonstressed state, a H3S10ph-associated factor (referred as “X factor”) prevents HDAC1/2/3 from accessing H4K5ac/K8ac for deacetylation and hence locks up the majority of Brd4 onto chromatin (Figure 2(a)). During stress response, the PP1*α* pathway dephosphorylates H3S10ph, and the “X factor” departs from the nucleosome. This allows stress-activated HDAC1/2/3 to access and deacetylate H4K5ac/K8ac, thereby releasing chromatin-bound Brd4 for subsequent stimulation of inducible gene expression (Figure 2(b)). In this context, the dephosphorylation of H3S10ph governs Brd4's role switching from chromatin targeting to regulating inducible gene expression.

For more than two decades, the signal-induced phosphorylation of H3S10 has been regarded as a positive epigenetic mark for transcriptional activation of inducible genes [96–100]. In contrast, we found that the dephosphorylation of H3S10ph is the key to Brd4-mediated expression of inducible genes ([64] and unpublished data). Although apparently contradictory, these events are likely to occur at different regions of the genome. We discover that, despite a significant decrease in global H3S10ph during stress treatments, there is an increased chromatin binding of 14-3-3 (unpublished data), an H3S10ph-associated protein that functions as a scaffold for recruiting chromatin remodeling factors [78, 100]. This result indicates that 14-3-3 and Brd4 are associated with different nucleosomes, and hence 14-3-3 is unlikely to be the “X factor”. Moreover, signal-induced H3S10 phosphorylation usually correlates with the chromatin remodeling of promoter [97], which occurs prior to transcription initiation. However, we observed that stress-induced H3S10ph dephosphorylation enables Brd4 to augment transcription elongation [64], an event subsequent to initiation. Hence, one may envision that for a small fraction of nucleosomes located at the promoter or enhancer regions of inducible genes, the signal-induced H3S10 phosphorylation facilitates chromatin remodeling for subsequent transcription initiation, whereas the global decline in H3S10ph may have distinct function in governing Brd4's release for subsequent P-TEFb recruitment and transcription elongation. As Brd4 represents a novel and promising drug target for cancer therapy, it is crucial to thoroughly elucidate its role in cell cycle progression and in epigenetic and transcriptional regulation.

## 3. HEXIM1

HEXIM1 was first identified in 1999 and the potential involvement of HEXIM1 in differentiation and cardiac development was suggested [101, 102]. However, the major biological function of HEXIM1 as the inhibitor of P-TEFb was revealed four years later by two research groups led by Olivier Bensaude and Qiang Zhou [29, 30]. Recent evidence demonstrated a role of HEXIM1 in cancers and regulation of the p53 pathway through the P-TEFb-dependent and -independent mechanisms [38, 103].

### 3.1. HEXIM1 as a Potential Tumor Suppressor

The potential involvement of HEXIM1 in cancers was first reported by Montano and coworkers in 2003. They identified HEXIM1 as a novel binding protein of estrogen receptor *α* (ER*α*) in a yeast two-hybrid screen using a cDNA library of MCF7 breast cancer cells. Since estrogens downregulated HEXIM1 expression at both protein and mRNA levels, HEXIM1 was also named estrogen downregulated gene 1 (EDG1) [104]. Estrogens are known to play a critical role in the growth of breast cancers and exert their effects by binding to their specific nuclear receptors. The ligand-bound ERs function as a transcriptional activator and upregulate several genes required for cell proliferation, such as cyclin D1 and C-MYC [105, 106].

ER*α* has been widely targeted in breast cancer therapy since it is present in more than half of breast tumors [107, 108]. Therefore, a potential role of HEXIM1 in regulating ER*α* in breast cancers was suggested. Breast cancer cells exhibited lower HEXIM1 expression when compared to normal breast epithelial tissue, and overexpression of HEXIM1 inhibited growth of both normal and breast cancer cells [104]. The molecular mechanism of cell growth inhibition by HEXIM1 was later elucidated. HEXIM1 interacted with ER*α* through its C-terminal region and inhibited the activity of ligand-bound ER*α* [109]. In addition, interaction between cyclin T1 and ER*α* was identified, suggesting the requirement of P-TEFb in regulating ER*α* activity during transcriptional activation [109]. Based on these findings, it was proposed that ER*α* might compete with HEXIM1 for binding to cyclin T1. Thus, the transcriptional activity of ER*α* depends on its association with P-TEFb (i.e., through the association with cyclin T1) and such a process is modulated by HEXIM1 [109].

Tamoxifen (Nolvadex), a selective ER modulator, has been used for more than 30 years to treat patients with advanced stage breast cancers [110]. However, tamoxifen resistance is a major problem in the treatment of ER-positive breast cancer patients. A recent study demonstrated that treatment with tamoxifen induced HEXIM1 recruitment to the promoters of ER target genes [111]. Since HEXIM1 was known to compete with cyclin T1 for binding to ER*α*, as expected, decreases in the abundance of cyclin T1 and serine 2-phosphorylated RNAPII on the coding regions of ER target genes were detected after tamoxifen treatment [111]. Furthermore, downregulation of HEXIM1 led to attenuation of the inhibition caused by tamoxifen on estrogen-induced gene expression and cell proliferation. Immunohistochemical studies using the samples of breast cancer patients showed that lower expression of HEXIM1 was associated with tumor recurrence in tamoxifen-receiving patients, suggesting that HEXIM1 is a critical determinant of tamoxifen resistance [111].

HEXIM1 is also involved in tumor progression. Angiogenesis is a physiological process governing the formation of new blood vessels from pre-existing vessels and an essential step in the transition of tumors from a benign state to a malignant one. Vascular endothelial growth factor (VEGF) functions as a major contributor to angiogenesis and its expression can be induced by estrogens (via ER*α*) or by hypoxia (via hypoxia inducible factor-1 alpha (HIF-1*α*)) [112]. HEXIM1 was found to regulate estrogen-induced VEGF transcription by inhibiting the recruitment of ER*α* to the VEGF promoter. Interestingly, HEXIM1 regulated this process in a P-TEFb-independent fashion [113]. Under hypoxic conditions, overexpression of HEXIM1 inhibited estrogen-induced expression of hypoxia-inducible factor-1 alpha (HIF-1*α*) protein and blocked the recruitment of HIF-1*α* to the promoter region of the VEGF gene [113]. HEXIM1 was later found to directly interact with HIF-1*α* and increase ubiquitination of HIF-1*α*, resulting in downregulation of HIF-1*α* protein expression [114].

Metastasis is the spreading of cancer cells from one organ or tissue to another. The development of metastases and angiogenesis are intrinsically connected [115]. The involvement of HEXIM1 in angiogenesis suggests a potential role of HEXIM1 in metastasis. A lower expression level of HEXIM1 was detected in metastatic breast cancers when compared with matched primary breast tumors [116]. Overexpression of HEXIM1, either by transgene expression or HMBA treatment, significantly inhibited metastasis and angiogenesis [116]. In addition, knockdown of HEXIM1 stimulated the invasion of MCF7 cells [116]. Taken together, these results suggest the tumor suppressor function of HEXIM1.

### 3.2. Regulation of HEXIM1 by NPM and MDM2

Results obtained from mass spectrometry in search for novel HEXIM1 binding proteins have led to the discovery of a functional interaction between HEXIM1 and the p53 pathway. Nucleophosmin (NPM; encoded by the *NPM1* gene), a nucleolar protein and a key regulator of p53, was identified as a HEXIM1 binding partner [117]. NPM enhances p53 activity directly by binding to and stabilizing p53 or indirectly by stimulating the induction of p53 through binding to other p53 regulators, such as HDM2 and ARF [118–121]. Mutation of the *NPM 1* gene is the most frequent mutation in acute myeloid leukemia (AML). About 35% of AML patients carrying NPMc+, the cytoplasmic-mislocated mutant form of NPM, indicate the pathological significance of this gene [122]. A distinct gene expression profile in NPMc+ AML cells was reported in an early study [123]; however, the connection between NPM mutation and transcriptional regulation remained to be elucidated.

We found that overexpression of NPM resulted in proteasome-dependent degradation of HEXIM1 and activation of P-TEFb [117]. This result demonstrated the functional significance of the HEXIM1-NPM interaction. The interaction between HEXIM1 and NPMc+ was also detected by immunoprecipitation (IP) [117]. In addition, using a green fluorescent protein (GFP) tagged NPMc+ fusion protein, immunofluorescence studies demonstrated that GFP-NPMc+ sequestered a portion of HEXIM1 in the cytoplasm [117]. Such mislocalization of HEXIM1 in the cytoplasm would have a significant impact on the equilibrium between active and inactive P-TEFb complexes in the nuclei by increasing the amounts of active P-TEFb complexes. In agreement with this observation, the cytoplasmic localization of HEXIM1 was only detected in an AML cell line carrying the NPMc+ mutation (i.e., AML3 cell line) [117]. Furthermore, comparing the AML cell line with wild-type NPM, lower HEXIM1 protein level was detected in AML3 cells [124]. As expected, an increase in P-TEFb-mediated transcription was detected in this NPMc+ AML cell line [117]. Collectively, these findings demonstrate a role for the NPMc+ mutation in transcriptional regulation and suggest the potential involvement of HEXIM1/P-TEFb in the tumorigenesis of AML.

**Figure 3:**
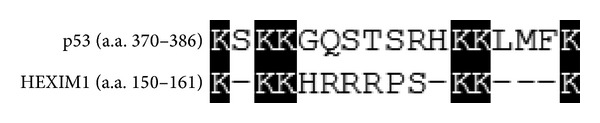
Alignment of the HDM2 ubiquitination sites between p53 (amino acids 370–386) and HEXIM1 (amino acids 150–161). The HDM2-ubiquitinated lysine residues are indicated in black boxes.

MDM2 (or HDM2, the human homolog) is best-known as the negative regulator of p53. HDM2 ubiquitinates p53 and leads to proteasome-mediated degradation of p53 [125, 126]. Six lysine residues, Lys-370, -372, -373, -381, -382, and -386, located within the C-terminal negative (NEG) domain of p53 are the target residues of HDM2 ubiquitination [127] (Figure 3). Recently, HEXIM1 was identified as a novel substrate of HDM2. Unexpectedly, the ubiquitination of HEXIM1 by HDM2 does not lead to proteasome-dependent degradation but still affects HEXIM1's function [128]. Compared to wild-type HEXIM1, ubiquitinated HEXIM1 exhibits a stronger inhibitory effect on P-TEFb activity, suggesting a potential role for HDM2 on regulation of P-TEFb [128]. Six lysine residues located in the middle region of HEXIM1 were identified as the major sites of HDM2 ubiquitination [128]. Sequence alignment of the ubiquitination sites between p53 (amino acids 370–386) and HEXIM1 (amino acids 150-161) exhibits similar distribution of the lysine residues (Figure 3), raising the possibility that HEXIM1 may compete with p53 in binding to HDM2 and regulate the stability of p53.

### 3.3. HEXIM1 as a Positive Regulator of p53

**Figure 4:**
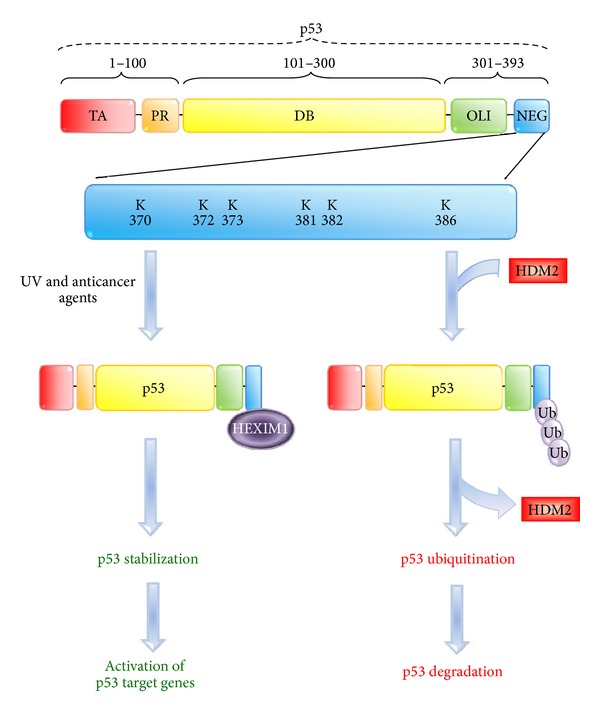
HEXIM1 stabilizes p53 by blocking the HDM2-mediated ubiquitination of p53. Human p53 protein can be divided into five domains: transactivation (TA), proline rich (PR), DNA binding (DB), oligomerization (OLI), and negative (NEG) domains. The HDM2 ubiquitination sites are located with the NEG domain. HEXIM1 interacts with the NEG domain of p53 and prevents the binding between p53 and HDM2.

Associations between HEXIM1 and two p53 regulators, NPM and HDM2, suggest the possible involvement of HEXIM1 in the p53 pathway. Indeed, the interaction between endogenous HEXIM1 and p53 proteins was detected by IP. The direct binding between HEXIM1 and p53 was demonstrated by an *in vitro* GST pull-down assay using the purified recombinant proteins [129]. Overexpression of HEXIM1 upregulated the expression levels of p53 and p53 target genes by blocking p53 ubiquitination mediated by HDM2 [129]. This was further supported by domain study, showing that HEXIM1 is bound to p53 through the NEG region of p53 (Figure 4). As stated earlier, the NEG domain contains six lysine residues ubiquitinated by HDM2 [127] (Figure 4). This helps to explain that through the interaction with the NEG region, HEXIM1 prevents p53 ubiquitination by HDM2 and enhances the stability of p53. In addition, it was noted that p53 interacted with the 7SK snRNA-free HEXIM1, indicating that HEXIM1 might positively regulate p53 in a P-TEFb-independent manner [129].

Importantly, HEXIM1 is an essential factor that regulates the induction of p53. UV radiation or treatments with the anticancer agents, such as doxorubicin, etoposide, flavopiridol, roscovitine, and nutlin-3, are known to induce and activate p53. In all conditions examined, elevated protein levels of p53 were found to associate with the increased p53-HEXIM1 interaction [129]. In contrast, knockdown of HEXIM1 completely blocked p53 induction and released the cell cycle arrest caused by p53 [129]. These findings reveal a novel role of HEXIM1 in the activation of p53 induced by anticancer agents and may lead to potential development of new anticancer strategies. As the requirement of p53 for angiogenesis and metastasis is wellestablished, it is possible that HEXIM1 inhibits tumor progression through activation of p53.

## 4. Conclusion

Involvement of Brd4 and HEXIM1 in tumorigenesis through the P-TEFb-dependent and -independent mechanisms opens a new and exciting venue for cancer research. Development of small molecules or other strategies to block the chromatin-binding of Brd4 or to induce the expression of HEXIM1 may provide novel therapeutic options against cancer.
